# When Ingroups Aren’t “In”: Perceived Political Belief Similarity Moderates Religious Ingroup Favoritism

**DOI:** 10.1371/journal.pone.0050945

**Published:** 2012-12-12

**Authors:** Carlee Beth Hawkins, Brian A. Nosek

**Affiliations:** Department of Psychology, University of Virginia, Charlottesville, Virginia, United States of America; George Mason University/Krasnow Institute for Advanced Study, United States of America

## Abstract

Motivated thinking leads people to perceive similarity between the self and ingroups, but under some conditions, people may recognize that personal beliefs are misaligned with the beliefs of ingroups. In two focal experiments and two replications, we find evidence that perceived belief similarity moderates ingroup favoritism. As part of a charity donation task, participants donated money to a community charity or a religious charity. Compared to non-religious people, Christians favored religious charities, but within Christians, conservative Christians favored religious charities more than liberal Christians did. Experiment 2 demonstrated that the perceived political beliefs of the charity accounted for the differences in ingroup favoritism between liberal and conservative Christians. While reporting little awareness of the influence of ideology, Christian conservatives favored religious charities because they perceived them as conservative and liberal Christians favored the community charity because they perceived it as liberal.

## Introduction

When many options are available, people tend to gravitate toward those associated with their identities. For example, one might expect Billy Graham, but not Richard Dawkins, to contribute money to a religious charity. This is because they have different social identities – Graham is a “Christian” and Dawkins is an “atheist”. In many cases, identifying with one’s social group leads to favoring the “ingroup” and its members, a robust phenomenon known as ingroup favoritism [1]–[3].

Religious group membership leads to ingroup favoritism. Verkuyten [4] found that Turkish Muslims in the Netherlands showed significant ingroup favoritism for their Muslim ingroup. Protestants who took part in the General Social Survey in the U.S. reported feeling more warmth toward other Protestants than toward Jews and Muslims [5]. Further, Hunter [6] experimentally manipulated the religious affiliation of a character in a series of vignettes to be either Christian or atheist. The Christian sample liked the person more if he was a member of their religious group.

Though ingroup favoritism is pervasive, individuals vary in the extent to which they show favoritism toward their groups. One variable accounting for these differences is political ideology – conservatives show stronger ingroup favoritism than liberals do across a variety of group memberships [7]–[9]. Graham, Haidt, and Nosek [10] demonstrated that conservatives endorse ingroup loyalty as a moral value more than liberals do. Further, social dominance orientation [11] – an individual trait representing preference for social hierarchy and group dominance – has been described as “a primary motivating force behind political conservatism” ([12] p. 478), and is positively related to ingroup favoritism [13].

This evidence suggests a general conclusion: liberal and conservative Christians should favor religious ingroups, with the effect being stronger among conservative Christians. However, religious group identities are special group identities because they provide a network of shared beliefs and values that are particularly explanatory and unique [14]. Therefore, religious group favoritism implies more than just identification with a religious group, but also endorsement of a particular set of beliefs. Religion is associated with conservatism in general and, in the United States, the Republican Party in particular [15]–[18], but not perfectly [19]–[20]. If liberal Christians perceive their religious ingroups to be more conservative than their personal ideology, it sets the stage for a conflict between beliefs and identity. A conservative Christian and a liberal Christian sharing the same strength of identification with their religious and political identities might still differ in their religious ingroup favoritism because of differences in perceived belief similarity. We investigated whether the political variation in ingroup favoritism was particularly pronounced in the context of religious groups and predicted that it would be mediated by the perceived political belief similarity between the self and the religious group.

### Belief Similarity as a Predictor of Ingroup Favoritism

While there is a tendency for people to adopt the beliefs of their ingroups [3], [21]–[22], and to project their own beliefs onto their ingroups [23]–[25], neither of these processes is comprehensive (see [26] for an empirical test between these two processes). People can recognize a distinction between themselves and their groups in regard to their personal beliefs. Optimal Distinctiveness Theory [27], for example, even suggests that individual uniqueness may be a motivational counterweight to the affiliative processes that bind people to their group memberships. Religious groups are associated with conservatism, so liberal Christians may perceive that their personal and group beliefs are misaligned, even if the conditions might normally result in favoritism.

Existing evidence supports our hypothesis that perceived belief similarity contributes to ingroup favoritism. People tend to favor ingroups that share similar beliefs more than ingroups that do not. In a minimal group context, where groups are created arbitrarily for the purposes of a study, Allen and Wilder [28] found that when individuals were led to believe that their ingroup shared similar attitudes to themselves, they showed higher ingroup favoritism than when the ingroup had mostly dissimilar attitudes. However, in this minimal context, there was still *some* favoritism for the ingroup even when it had dissimilar attitudes. Chen and Kenrick [29] found that attitude similarity of ingroup and outgroup members affects liking of that individual group member. Democrats and Republicans were introduced to a member of their party (ingroup) or the other party (outgroup), and led to believe that the person had similar attitudes and beliefs (e.g., a Democrat with liberal beliefs for a Democratic participant) or dissimilar beliefs (e.g., a Democrat with relatively conservative beliefs for a Democratic participant) and then made judgments about the target person. Ingroup members are expected to have similar beliefs, so when people discover that ingroup members have dissimilar beliefs, these members are disliked. However, outgroup members (e.g., Republicans for Democratic participants) were not given this same penalty for misaligned beliefs. Extrapolating individual attraction to a group favoritism context, these findings suggest that liberals who perceive their religious ingroup to have dissimilar beliefs will demonstrate decreased favoritism for the group.

Additional evidence for the role of belief similarity in predicting favoritism comes from Sani and Todman’s [30] schism model, which holds that group members who view the group as having violated a fundamental group value will view their identity as subverted. These group members feel that they have lost their voice and view the group as less cohesive. For liberal Christians, the link between religion and conservatism may be perceived as a violation of their values, and they may question their commitment to their religious groups. In the schism model, these disenfranchised group members may leave the group to form a subgroup, or join an existing subgroup [30]. For example, it appears that schisms occurred in the Church of England when the policy allowing women to be ordained was enacted [31] and in the Italian Communist Party after announcements of political realignment [32]. Further, Glasford and colleagues [33]–[34] theorize that when ingroups violate important personal values, individuals experience intragroup dissonance – psychological discomfort resulting from disagreement between self and ingroup. When Americans perceived that their ingroup (United States) violated an important personal value (providing health care to American citizens), they experienced psychological discomfort and disidentified with their American ingroup [33].

The above research suggests an interplay between identification and belief similarity, but also suggests that if Christian liberals perceive belief dissimilarity with their religious ingroups, they would simply leave the group. Some liberal Christians may indeed switch to more liberal religious denominations or dissolve their religious identities entirely when faced with belief dissimilarity [15]. Our perspective incorporates such a possibility, but recognizes that leaving religious groups can be difficult. Similar to cultural memberships or marriages, religious identities are reinforced by a number of processes outside of shared beliefs. Liberal Christians may remain strongly identified with their religion despite belief dissimilarity because of status quo bias [35], public or private commitment to the identity regardless of other circumstances, social support and networks provided by the identity, family expectations or commitments, and history effects (“I grew up in this church, how could I leave it?”). Further, Ysseldyk, Matheson, and Anisman [14] suggest that religious group identification is an eternal group membership. Beliefs in higher powers and the afterlife that are present in many religions imply that one may *never* leave the social group. The panoply of factors that can affect identification with groups introduces the possibility that belief similarity may instead play a role in the operation of how much religious groups are favored (leaving identification constant, or at least influencing it less). In our case, we hypothesize that regardless of the strength of identification with the religious ingroup, liberal Christians will show less favoritism for their religious groups than conservative Christians because they perceive dissimilarity between their personal beliefs and those of the religious ingroup.

### With What Group are People Identifying?

People can define their religious identities in a variety of ways. For example, a Methodist could describe him/herself as religious, Christian, or Methodist. Each of these religious labels describes membership in the “religious” group, but they vary in how general or specific they are. To test whether our hypothesis applies generally across conceptions of one’s religious ingroup, we investigated whether framing of the ingroup influenced the extent to which the group was favored. Further, explicitly manipulating the framing of the group allows us to address a potential alternative explanation for differences in ingroup favoritism between liberals and conservatives – that they think of their religious group identity at different levels of specificity. For example, liberal Roman Catholics could avoid considering belief dissimilarity with their specific religious ingroup by framing their group membership in more general terms. This manipulation allows us to examine potential variation in identification when we manipulate how the ingroup is represented.

## Experiment 1

We tested whether political ideology moderates religious ingroup favoritism. Because religion is associated with conservatism, we expected that political ideology would moderate favoritism and that liberals would resist favoring religious ingroups. Following common operationalizations, favoritism was tested in a monetary allocation paradigm in which people simulated donating money to a variety of charities (e.g., [1]). The key manipulated charity was either denoted as religious or did not mention religion (herein known as the ‘secular’ charity framing). Group identification and favoritism research has focused heavily on intergroup evaluations. However, Brewer [36]–[37] argued that intergroup bias consists of two processes: ingroup favoritism and outgroup derogation. While these two processes can be related, they are not fundamentally dependent. In this vein, the current research investigates ingroup favoritism *independent* of a salient outgroup. Because we are interested only in how people favor ingroups and not whether they favor ingroups relative to outgroups, our comparison is religious ingroup identity compared to a general group identity, the community. For this reason, the current research is arguably a more stringent test of ingroup favoritism than comparing religious groups to an outgroup.

To examine whether religious groups are differentially favored by conservatives and liberals, we recruited two samples: Christians and non-religious people. We manipulated whether participants contributed money to a secular charity, a religious charity, a Christian charity, or a charity characterized as the participant’s specific denomination. Comparing Christians to non-religious people enables a comparison of the distinct contributions of religious group membership and political ideology on favoritism. Overall, because they are outgroups, we expected non-religious people to give less money to religious charities than Christians did. Comparing liberal Christians and conservative Christians allowed us to determine whether political ideology influenced favoritism *within* the religious ingroup. We expected conservative Christians to favor the religious (ingroup) charities over the secular charity, but for liberal Christians to show weak favoritism, or none at all.

### Experiment 1 Method

#### Participants

Fifteen hundred ninety-one (63% female) volunteers provided electronic consent and completed the study on the Project Implicit research website (https://implicit.harvard.edu). The study was approved by the University of Virginia Institutional Review Board. Demographics, including religion, were collected during registration. Participants were unobtrusively selected for this study from a pool of possible studies based on religious affiliation. Only participants who completed the key dependent variable (contribution) were retained. Participants who consented and completed the study to debriefing (*n* = 1401) did not differ from noncompleters (*n* = 190) on education, *t*(1587) = 0.31, *p* = .756, *d* = 0.02, gender, χ^2^(1, *N* = 1591) = 2.13, *p* = .145, *Phi* = 0.037, age, *t*(1587) = 0.86, *p* = .388, *d* = 0.04, or political ideology, *t*(1557) = −0.06, *p* = .955, *d* = 0.00.

#### Christians

The Christian group consisted of 1073 participants (M_age_ = 32.32, 66% female) from four major Christian denominations: 178 (17%) Baptists, 104 (10%) Lutherans, 144 (13%) Methodists, and 647 (60%) Roman Catholics. These denominations were chosen because they were the largest Christian denominations represented in the research pool. The modal education level was “some college” and ethnicity was 81% non-Hispanic or Latino, 12% Hispanic or Latino, and 8% unknown. The racial composition of the sample was 79% White, 7% Black or African American, 6% mixed race, and 8% other or unknown.

#### Non-religious people

Non-religious people were those who identified themselves as agnostic (*n* = 156), atheist (*n* = 172), deist or theist (*n* = 8), other non-religious (*n* = 75), spiritual, no organized religion (*n* = 87), or none (*n* = 20). We removed seven people who reported (a) not belonging to a religion during registration and (b) being “very religious” or that religion was an “extremely important” group to which they belonged when they completed the study suggesting lack of attention or motivation. The non-religious group included 518 participants (M_age_ = 30.45, 57% female) whose modal education level was “bachelor’s degree” and ethnicity was 85% non-Hispanic or Latino, 6% Hispanic or Latino, and 9% unknown. The racial composition of the sample was 83% White, 3% Black or African American, 7% mixed race, and 8% other or unknown. Christians were more likely to be female (66%) than NRs (57%), χ^2^(1, *N* = 1591) = 14.12, *p*<.001, *Phi* = 0.094. Christians were less likely to be White (78%) than NRs (86%), χ^2^(1, *N* = 1498) = 12.90, *p*<.001, *Phi* = 0.093.

### Materials

#### Political ideology and religiosity

Political ideology was measured with a single item asking people to report how liberal or conservative they were on a 7-point scale ranging from −3 (*Strongly conservative*) to 3 (*Strongly liberal*). Religiosity was measured with a single item asking people to report their religiosity on a 4-point scale ranging from 1 (*Not at all religious*) to 4 (*Very religious*). Both items have been used effectively as simple measures of political ideology and religiosity [9], [38]–[39].

#### Social identity questionnaire

To assess the relative importance of different social identities, participants reported how important each of the following was to their lives: occupation, country, political party, religion, and age. Responses were recorded on a 5-point scale ranging from 1 (*Not at all important*) to 5 (*Extremely important*) with a *Doesn’t apply to me* option for occupation, political party, and religion. This questionnaire was adapted from similar scales that have been used to measure social identity in a variety of contexts (see [40] for an overview of social identity measures).

#### Charity donation task

Participants were instructed to imagine they had $1000 to donate to charities and divided the money amongst six charities. Five were filler charities and were acquired from a charity donation website [41]: AAA Foundation for Traffic Safety: *Dedicated to ensuring road safety,* Defenders of Animal Rights: *Dedicated to stopping animal cruelty,* Galapagos Conservancy: *Preserving a world treasure,* Institute for Educational Advancement: *Supporting education of our nation's youth,* and Diabetes Research Institute Foundation: *Seeking a cure*. The charities were chosen because they were real but likely to be unfamiliar, and were not obviously political or religious. The number of dollars contributed to all the charities was automatically calculated and displayed on the screen. Participants could not proceed to the next part of the study until exactly $1000 was allotted.

The name of the sixth charity, the “Community Service Center”, was invented and manipulated between participants as the “Community Service Center,” “Religious Community Service Center,” “Christian Community Service Center,” or the specific Christian denomination of the participant. For example, it was “Baptist Community Service Center” for Baptists who were randomly assigned to the specific denomination condition. Non-religious participants randomly assigned to the specific denomination condition were again randomly assigned to any one of the four Christian denomination labels (Baptist, Lutheran, Methodist, Roman Catholic). The description of the Community Service Center, *serving the less fortunate,* remained constant across all experimental conditions, regardless of whether it was described as religious or not.

### Procedure

Participants completed all demographics upon registration at the research site, including political ideology and religiosity. Minutes to months later they logged in to complete a study and were randomly assigned to this one. Once assigned, they completed the social identity questionnaire and charity donation task in a randomized order. After completing the social identity questionnaire and charity donation task, participants completed an Us-Them Implicit Association Test [42] that is not relevant for the current report.

### Analysis Strategy

The charity framing manipulation (denoting religious ingroup or not) was coded to allow for estimation of regression coefficients in a generalized linear model: secular (*0*) and religious (*1*). Participants’ (measured) religious group membership was coded similarly: non-religious (*0*) and Christian (*1*). The secular framing of the key charity (Community Service Center) served as the “secular” level, and each religious framing of key charity (“Religious Community Service Center”, “Christian Community Service Center”, and the specific denomination charities) was collapsed for the main analyses to form the “religious” level, but each was tested separately in follow-up tests. Self-reported political ideology served as a (measured) continuous independent variable (−3 *strongly conservative* to 3 *strongly liberal*), and the dependent variable was contribution to the charity in dollars. Regression coefficients are unstandardized and reported with 95% confidence intervals, alongside sample statistics and significance tests, *b*, *CI* = 2.5%, 97.5%, *t*(df), *p*. Positive regression coefficients reflect higher contributions to the religious charities, by Christians compared to non-religious people, and by liberals compared to conservatives. Age and education sometimes covary with political ideology, so age, education, and their interactions with charity framing were covariates in all the models to ensure that any charity framing by political ideology interactions were independent of these variables. Analysis results were similar without the covariates in the models.

### Experiment 1 Results

#### Political ideology and religiosity

On average, the Christian sample was slightly more liberal than conservative (*M = *0.46, *SD = *1.63) and near the midpoint on the religiosity item (*M = *2.44, *SD = *0.84). As expected, the non-religious sample (NRs) was not religious (*M = *1.18, *SD = *0.43) and was politically liberal (*M* = 1.52, *SD = *1.35).

#### Political ideology and religious group membership predicted contribution

Table 1 summarizes the average contribution to each of the six charities and their correlation with political ideology. To test whether political ideology moderated the degree of favoritism for religious ingroups, we entered charity framing (manipulated: secular or religious), political ideology (measured), and their interaction as predictors of contribution in a simultaneous regression. In the same model, we included religious group membership (measured: Christian or NR) and its 2-way interactions with charity framing and political ideology to investigate contribution across religious group membership (see Table 2 for a summary report of the regression results). The order of the charity donation task and the social identity questionnaire was manipulated, but had no effect on the results, so was not included in the final analysis. Centering predictor variables is standard practice for eliminating multicolinearity in continuous predictor variables, but political ideology has a rational zero point (moderate) that, when retained, facilitates interpretation of unstandardized regression coefficients in reference to ’moderate’. In this and all future analyses in this paper, effects are similar when political variables are centered on the group mean prior to analysis.

**Table 1:** Christians’ Contributions to Charities and Correlation with Political Ideology for Experiments 1 and 2.

	Experiment 1			Experiment 2			
Charity: *Description*	$$ contributed		*r* with politics	$$ contributed		*r* with social politics	
AAA Foundation for Traffic Safety: *Dedicated to ensuring road safety*	67	−.11		74	−.04		
Community Service Center: *Serving the less fortunate*	233	−.07		232	−.14		
Defenders of Animal Rights: *Dedicated to stopping animal cruelty*	130	.03		147	.09		
Galapagos Conservancy: *Preserving a world treasure*	102	.11		88	.15		
Institute for Educational Advancement: *Supporting education of our* *nation’s youth*	283	.11		278	.04		
Diabetes Research Institute Foundation: *Seeking a Cure*	185	−.11		182	−.07		

*Note.* Total Contribution = $1000 without rounding error. All correlations are significant at the *p*<.05 level except the Community Service Center and Defenders of Animal Rights in Experiment 1 and AAA Foundation for Traffic Safety and Institute for Educational Advancement in Experiment 2.

**Table 2:** Regression Results for Experiment 1.

Model Term	Unstandardized coefficient	*p* value of *t* statistic
Charity framing	17.67	.728
Political ideology	17.66	.024
Religious group membership	45.50	.047
Charity framing*Political ideology	−28.67	<.0001
Charity framing*Religious group membership	81.68	.001
Political ideology*Religious group membership	−5.82	.383
Age	2.91	<.0001
Education	7.23	.322
Age*Charity framing	−1.89	.028
Education*Charity framing	−8.46	.315
Charity framing*Political ideology*Religious group membership	−3.58	.822

*Note.* The 3-way interaction was tested and dropped from the model, so the model reported in the text has only main effects and 2-way interactions. Political ideology was measured on a scale of −3 (*strongly conservative*) to 3 (*strongly liberal*). Charity framing was dummy coded as community (*0*) or religious (*1*) and religious group membership was dummy coded as nonreligious (*0*) or Christian (*1*). Unstandardized regression coefficients should be interpreted in the context of these scales.

#### Religious charities were favored by Christians

A main effect emerged for religious group membership, *b* = 45.50, *CI* = 0.65, 90.35, *t*(1546) = 1.99, *p* = .047, such that Christians gave more overall to the key charity than NRs did. This main effect was qualified by an interaction between religious group membership and charity framing, *b = *81.68, *CI* = 34.74, 128.61, *t*(1546) = 3.41, *p*<.001. Follow-up tests revealed that Christians (*M* = $251) and NRs (*M* = $231) did not differ in their contribution when the charity was framed as secular, *t*(381) = −0.95, *p = *.341, *d* = −0.10, but Christians (*M* = $226) gave more than NRs (*M* = $90) when the charity was framed as religious, *t*_satterthwaite_ (1092.4) = −14.59, *p*<.0001, *d* = −0.88. This demonstrates ingroup favoritism by Christians relative to NRs – Christians contributed more money to the religiously framed charities.

#### Political ideology moderated favoritism

**Figure 1:**
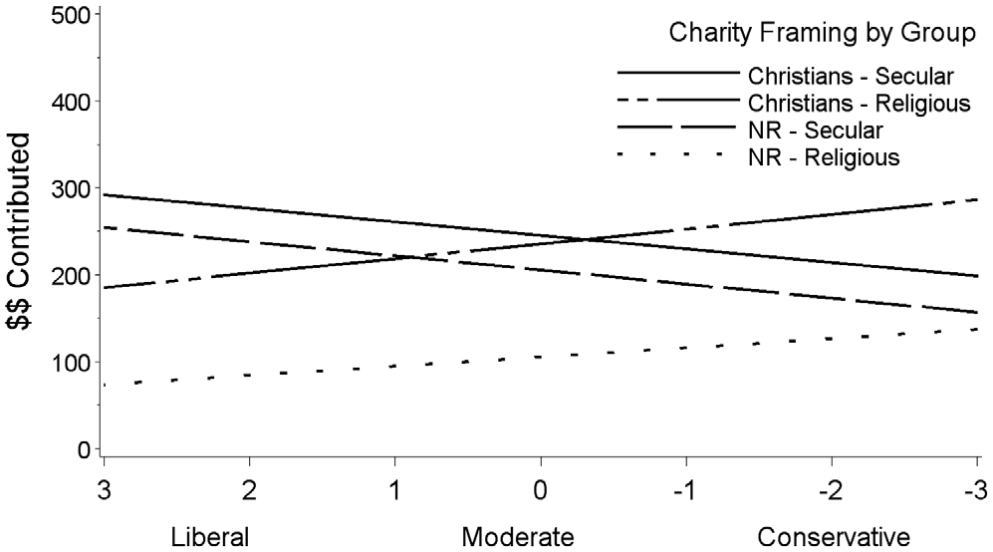
Political ideology predicts favoritism for both Christians and nonreligious people. Regression analysis predicting the number of dollars contributed to the Community Service Center Charity by political ideology and charity framing (community or religious) by both Christians and non-religious people for Experiment 1.

A main effect emerged for political ideology, *b* = 17.66, *CI* = 2.28, 33.04, *t*(1546) = 2.25, *p* = .024, such that liberals contributed more to the key charity regardless of whether it was framed as secular or religious. As predicted, a significant 2-way interaction between political ideology and charity framing qualified this main effect, *b* = −28.67, *CI* = −41.93, −15.42, *t*(1546) = −4.24, *p*<.0001. To investigate the interaction, we calculated simple regressions between political ideology and contribution for secular versus religious charity framing. Liberals tended to give slightly more money to the key charity than conservatives did in the secular framing condition, *b*(372) = 12.15, *p* = .042, *r* = .11, and less than conservatives in the religious framing condition, *b*(1183) = −25.78, *p*<.0001, *r* = −.22. Figure 1 displays the regression lines separately for liberals and conservatives for both NR and Christian samples.

To ensure that these results were not driven by members of just one of the four Christian denominations, we tested whether religious denomination moderated the interaction between political ideology and charity framing. The 3-way interaction between charity framing, political ideology, and religious denomination was not significant, *F* (3, 1033) = 0.83, *p = *.477, in this study and in Experiment 2, *F* (3, 875) = 0.16, *p = *.926, suggesting that denomination does not qualify the reported results.

#### Christians and NRs displayed the same pattern of contribution

To determine whether political ideology moderated contribution differently for Christians and NRs, we tested the 3-way interaction between charity framing, political ideology, and religious group membership, and this was not significant, *b* = −3.58, *CI* = −34.80, 27.65, *t*(1545) = −0.22, *p* = .822. The lack of 3-way interaction suggests that the observed effects of charity framing and political ideology on contribution do not depend on whether the participant is a member of the religious ingroup or not. Both Christians and NRs are perhaps using their perceived belief similarity, rather than their group membership, to inform whether they should contribute to the key charity.

#### Possible alternative explanations

Within the Christian group, differences in the level of identification with the religious group or the levels of framing the religious charity may also influence favoritism, perhaps qualifying our interpretation of the above findings. NRs are not relevant for these alternative explanations, so the next two analyses are conducted on the Christian sample only.

#### Differences in religious importance across the political spectrum

Perhaps not surprisingly, given the association between religion and conservatism, conservative Christians reported that their religious groups were more important to them (*M* = 3.69, *SD* = 1.22) than liberal Christians did (*M* = 2.82, *SD* = 1.21), *t*(703) = 9.02, *p*<.0001, *d* = 0.68. To test whether variation in religious group importance moderated charitable contributions, we added religious group importance (centered) and its interactions with charity framing and political ideology to the regression model. The interaction between political ideology and charity framing remained significant, *b* = −27.12, *CI* = −43.23,−11.02, *t*(1038) = −3.31, *p = *.001, and the interaction between religious group importance and charity framing was not a significant predictor of contribution, *b* = 16.80, *CI* = −3.84, 37.44, *t*(1038) = 1.60, *p = *.111. Differences in degree of religious group importance between liberals and conservatives did not account for the political differences in religious group favoritism. This demonstrates that even though liberal and conservative Christians may value their religious group identities to different degrees, this difference in religious group importance does not predict how much they favor their religious ingroups.

#### Levels of framing the religious ingroup

To investigate the possibility that the observed effects might only hold for specific framings of the religious ingroup (religious, Christian, specific denomination), we repeated the analysis above three times on the Christian sample. In each analysis, the religious charity framing was coded as *1* and the secular charity framing as *0*. The charity framing by political ideology interaction was replicated for the ‘religious’ framing, *b* = −33.31, *CI* = −53.42, −13.20, *t*(526) = −3.25 *p* = .001, Christian framing, *b* = −15.52, *CI* = −34.97, 3.92, *t*(514) = −1.57, *p* = .118, and denomination framing, *b* = −39.20, *CI* = −59.71, −18.68, *t*(513) = −3.75, *p*<.001, though not reliably for the Christian framing. While in the right direction, this suggests some caution when interpreting the political differences when the religious charity is framed as “Christian”. To be confident that the effect is general across framing, we conducted two replications (reported after the Experiment 1 discussion) and then tested it again in Experiment 2.

### Experiment 1 Discussion

Conservative Christians gave more money than liberal Christians did to a charity that was explicitly identified as religious compared to one that was not. Further, liberal Christians did not favor religious ingroups more than a secular group despite self-identifying as Christian. Since religion is associated with conservatism, conservatives may perceive more belief similarity between the self and the religious ingroup than liberals do. We pursue more direct evidence for this explanation in Experiment 2.

Christians contributed more money to the religious charities than non-religious people (NRs), so membership in the religious ingroup did predict favoritism. However, the patterns of giving for conservatives and liberals were similar across the religious ingroup sample (Christians) and the religious outgroup sample (non-religious people). Liberals, whether Christian or non-religious, favored the religious charities less than conservatives did. Moreover, conservative NRs gave more money to the religious groups than liberal NRs, demonstrating a similar pattern to conservative Christians. Calling this “outgroup favoritism” is not quite right, as both liberal and conservative NRs give considerably less to the religious groups than Christians. Instead, we interpret this finding as evidence that belief similarity between personal beliefs and group beliefs can influence contribution even when one does not belong to the group.

## Replication Studies

We conducted two studies replicating and extending the results of Experiment 1. Because their methodology is extremely similar to Experiment 1, we report them briefly. The first (*N = *842) was identical to Experiment 1 but one of the filler charities in the charity donation task was Partners in Health: *Providing health care for the uninsured*. This charity was overwhelmingly favored by liberals but not conservatives, and therefore may have differentially affected the attractiveness of the key charity. Even so, the findings demonstrated the same pattern as Experiment 1 – the interaction between political ideology and charity framing predicted favoritism, *b* = −16.96, *CI* = −34.96, 1.04, *t*(813) = −1.85, *p* = .065. Liberals contributed less to the key charity when it was framed as religious, *b*(616) = −26.23, *p*<.0001, *r* = −.23, whereas no significant differences between liberals and conservatives were observed when the key charity was framed as secular, *b*(203) = −7.13, *p* = .354, *r* = −.07.

A second replication (*N* = 1569) extended the findings of Experiment 1 and clarified the moderating role of political ideology on ingroup favoritism. Recent research suggests that so-called *social liberals/conservatives* and *economic liberals/conservatives* may differ in important ways. Libertarians, for example, are economic conservatives but they tend to be socially liberal. Haidt, Graham, and Joseph [43] demonstrated that Libertarians resemble secular liberals in their moral concerns about ingroup loyalty, and both are much less concerned about ingroup loyalty than are social conservatives. Additionally, where liberalism differs most from the conservative ideology of religious groups is in views on social issues, such as abortion and gay marriage. Our expectation was that stronger ingroup favoritism among conservatives than liberals was primarily driven by *social* political ideology, not *economic* political ideology. We used the same design and added two single-item responses measuring social and economic ideology following the format of that item in Experiment 1. In a sample of Christians, when social and economic political ideology were entered into the model simultaneously, the interaction between social political ideology and charity framing significantly predicted favoritism, *b* = −33.91, *CI* = −48.27, −19.55, *t*(1395) = −4.63, *p*<.0001, but the interaction between economic political ideology and charity framing did not, *b* = −0.60, *CI* = −16.27, 15.07, *t*(1395) = −0.07, *p* = .940. Social liberals gave less than social conservatives to the key charity when it was framed as religious, *b*(1060) = −20.36, *p*<.0001, *r* = −.21, and more when it was framed as secular, *b*(348) = 12.99, *p* = .008, *r* = .14.

To test the robustness of this effect across charity framing, we coded the secular framing as *0* and the religious framing as *1* for each level of the religious charity framing. In the first replication study, the interaction between political ideology and charity framing demonstrated the same trend across all framings of the religious group: religious (*p* = .055), Christian (*p* = .095), and denomination (*p* = .296). In the second replication study, all levels were significant: religious (*p*<.001), Christian (*p*<.0001), and denomination (*p*<.0001). The average effect size for the two main studies and the two replication studies was similar across the different framings: religious (*b* = −26.83), Christian (*b* = −21.96), denomination (*b* = −28.52), suggesting that the variation in significance across studies was random.

The first replication study demonstrates the robustness of political differences in religious group favoritism, even when distracter charities are quite appealing to one side of the political spectrum. This second replication study supports the results of Experiment 1 and the first replication study, showing that liberals give less to religious ingroups than conservatives. Further, this study extends these findings by demonstrating that social political ideology is responsible for this relationship, and not economic political ideology. Therefore, we focused on social political ideology as the key ideology moderator in Experiment 2.

## Experiment 2

The first study demonstrated that political ideology moderates favoritism for religious groups. The purpose of Experiment 2 was to identify why. We argued that because religious groups are associated with a particular belief system (political conservatism), this cues belief similarity for conservative Christians, but dissimilarity for liberal Christians, resulting in decreased ingroup favoritism. To test this, we measured the perceived political ideology of the key charity after the donation had been made and tested whether the perceived political ideology of the charity could account for the differential ingroup favoritism for religious groups between liberal and conservative Christians. Further, we tested whether participants were aware of this effect by asking them the extent to which they used the perceived political ideology of the key charity to guide their donation decisions. In Experiment 1, religious group membership was similarly salient across all participants, but in Experiment 2 we manipulated salience to measure whether belief similarity would cue favoritism across levels of religious group salience.

### Experiment 2 Method

#### Participants

Nine hundred eighty-one participants (M_age_ = 29.20; 72% female) volunteered and electronically consented to complete the study on Project Implicit. The study was approved by the Institutional Review Board at the University of Virginia. As in Experiment 1, Christian participants were unobtrusively selected for the study: 192 (20%) Baptists, 114 (12%) Lutherans, 123 (13%) Methodists, and 552 (56%) Roman Catholics. Non-religious participants were not recruited for this study. Participants’ modal education level was “some college” and the ethnic composition of the sample was 82% non-Hispanic or Latino, 11% Hispanic or Latino, and 7% unknown. The sample was 79% White, 9% Black or African American, 5% mixed race, and 7% other or unknown. Completers (*n* = 840) did not differ from noncompleters (*n* = 141) on gender, χ^2^(1, *N* = 980) = 0.270, *p* = .603, *Phi = *0.02, political ideology, *t* (962) = 0.43, *p* = .666, *d* = 0.03, or education, *t* (973) = −0.32, *p* = .750, *d* = −0.02. Completers (*M*_age = _28.74) were younger than noncompleters (*M*_age_ = 31.91), *t*_satterthwaite_ (177.9) = 2.61, *p* = .01, *d* = 0.39.

### Procedure

The charity donation task was identical to Experiment 1. The social identity questionnaire was replaced with the common and well-validated Collective Self Esteem Scale [44] adapted to measure religious identification and self-worth. Participants first completed either the charity donation task or the religious Collective Self Esteem Scale and then the political ideology questionnaire. The order of the charity donation task and the Collective Self Esteem Scale was manipulated to test whether salience of the religious group identity influenced favoritism. A perceived politics questionnaire, which measured how liberal or conservative participants viewed each charity, was completed last so that it would not make the perceived politics of the charities salient during the donation task. At the end of the study, participants were asked whether the perceived politics of the charities influenced their contribution choices. The political ideology questionnaire was counterbalanced with a political identification Implicit Association Test [10]. The IAT interacted with charity framing to predict favoritism similarly to self-reported political ideology in Experiment 1, *b* = −47.78, *CI* = −96.61, 1.04, *t*(852) = −1.92, *p* = .055, replicating the results with an implicit measure of political ideology. The order of the questionnaire and IAT had no significant effect on any analyses and will not be reported further.

### Materials

#### Collective self-esteem

Religious group identification was measured with a modified version of the Collective Self Esteem Scale. All items were edited to reflect identification with a religious group (e.g., *I am a worthy member of the groups that I belong to* was changed to *I am a worthy member of the religion that I belong to*). In an effort to minimize study session time, two representative items from each of the four subscales (membership, private, public, identity) were chosen. Responses were recorded on a 6-point scale ranging from 1 (*Strongly disagree*) to 6 (*Strongly agree*). *I’m not religious* was an additional option on the response scale. The items were averaged to form a single religious collective self-esteem score (α = .75).

#### Political ideology

Participants reported how liberal or conservative they were separately for social and economic issues on a 7-point scale ranging from −3 (*Strongly conservative*) to 3 (*Strongly liberal*). The first item measured political ideology concerning social issues, and provided the following examples of social issues: abortion, gay marriage, gun control. The second item measured political ideology concerning economic issues, and provided the following examples of economic issues: free market policies, taxation. An item measuring political party affiliation was also included: “Which political party best represents your beliefs?”: Democratic, Republican, Green, Libertarian, Other, or Don’t Know. This item was not included in the present analysis.

#### Perceived politics questionnaire

A single item for each of the six charities assessed participants’ perceived political ideology of all six charities in their study condition. Ratings were made on a 7-point scale ranging from −3 (*Strongly conservative*) to 3 (*Strongly liberal*). Participants also rated how much they were thinking about the perceived politics of the charities when they originally viewed them on a 5-point scale ranging from 1 (*Not at all*) to 5 (*A great deal*). Using a 5-point scale ranging from 1 (*No influence*) to 5 (*A great deal of influence*), participants rated the extent to which their perception of the political ideology of the charities influenced their contribution to the charities and to what extent it influenced their contribution to the key charity in particular.

### Experiment 2 Results and Discussion

#### Political ideology and religiosity

On average, participants were somewhat religious (*M* = 2.52, *SD* = 0.83). Participants were slightly more liberal than conservative (*M* = 0.20, *SD* = 1.63) and reported being more liberal on social issues (*M* = 0.40, *SD* = 1.93) than economic issues (*M* = −0.06, *SD* = 1.64). Social and economic political ideology were correlated, *r*(902) = .54, *p*<.0001, and both were strongly correlated with the single item political ideology measure from website registration,social: *r*(887) = .72, *p*<.0001; economic: *r*(888) = .71, *p*<.0001.

#### Social political ideology moderated favoritism regardless of religious identity salience

Charity framing was coded as a categorical variable as in Experiment 1 with the secular framing condition serving as the secular level (*0*) and the “religious”, “Christian”, and specific denomination charities serving as the religious level (*I*). Charity framing, social political ideology, and their interaction were used to predict favoritism. Replicating Experiment 1, the 2-way interaction between charity framing and social political ideology was significant, *b* = −23.77, *CI* = −37.83, −9.70, *t*(887) = −3.32, *p* = .001. To demonstrate that social political ideology was the key moderator, charity framing, social political ideology, economic political ideology, and their 2-way interaction terms were entered into a multiple regression as predictors of ingroup favoritism. The interaction between social political ideology and charity framing significantly predicted favoritism, even when economic political ideology was included, *b* = −18.73, *CI* = −35.91, −1.56, *t*(884) = −2.14, *p* = .033. However, the interaction between economic political ideology and charity framing was unrelated to ingroup favoritism, *b = *−*9.97, CI = *−29.81, 9.88, *t*(884)* = *−0.99, *p = .*325, when social political ideology was included.

Identity salience is known to influence favoritism [45]–[46], so one possible explanation for liberal Christians’ lack of favoritism for religious groups is that their religious identity was not salient and therefore did not elicit any religious group favoritism. To test this, we manipulated religious identity salience by presenting the Religious Collective Self-Esteem Scale before or after the donation task, and coded task order: *0* for charity donation task first and *1* for Religious Collective Self Esteem Scale first. Religious identity salience, social political ideology, and charity framing were used to predict favoritism in a multiple regression. The 3-way interaction was not significant, *b* = 4.91, *CI* = −23.06, 32.89, *t*(883) = 0.34, *p* = .730, suggesting that the salience of religious identity did not moderate the key result. Thus, we removed the salience variable from the model for the subsequent analyses.

#### Belief similarity accounted for the political variation in favoritism

Participants rated the secular charity as liberal (*M* = 1.28, *SD* = 1.46) and the religious charities as conservative (average across religious charities: *M* = −0.63, *SD* = 1.88). Table 3 reports the perceived political positions for each charity framing and the relationship between participants’ social political ideology and perceived politics of the charities. Despite some slight variation across charity framing and participant political ideology, in each charity framing condition, both conservatives and liberals perceived the secular charity as liberal and the religious charities as conservative.

**Table 3:** Perceived Politics of the Charities and Correlation with Political Ideology in Experiment 2.

Charity	Perceived politics	*r* of perceived politics with social political ideology
Community Service Center	1.28	.22
Religious Community Service Center	−0.40	.03
Christian Community Service Center	−0.71	.18
[Denomination] Community Service Center	−0.76	.05
Baptist Community Service Center	−0.91	.16
Lutheran Community Service Center	−0.28	.13
Methodist Community Service Center	−0.16	.04
Roman Catholic Community Service Center	−0.95	.00

*Note.* Perceived politics and social political ideology were measured on the same scale of −3 (*strongly conservative*) to 3 (*strongly liberal*). Each denomination charity was only viewed by participants of that denomination.

To test whether perceived belief similarity accounts for the effect of political ideology on religious ingroup favoritism, we added the main effect of perceived politics to the above regression model with charity framing, social political ideology, and their 2-way interaction. The charity framing by social political ideology interaction was significant, *b* = −24.46, *CI* = −39.40, −9.52, *t*(813) = −3.21, *p* = .001, and perceived politics significantly predicted favoritism, *b* = 9.04, *CI* = 2.32, 15.76, *t*(813) = 2.64, *p* = .008, – on average, more money was contributed if the charity was perceived as liberal. Next, we entered all main effects and 2-way interactions between perceived politics, charity framing, and social political ideology. Our key prediction was that the political variation in favoritism to secular and religious charities would be accounted for by differences in the perceived politics of the charities. Indeed, the interaction between perceived politics and social political ideology was a significant predictor of favoritism, *b* = 8.11, *CI* = 4.66, 11.56, *t*(811) = 4.62, *p*<.0001, and the original interaction between charity framing and social political ideology was rendered nonsignificant, *b* = −8.86, *CI* = −25.49, 7.78, *t*(811) = −1.05, *p* = .296, suggesting that perceived politics of the charity accounted for the political differences in favoritism. Table 4 reports the regression coefficients for these models.

**Table 4:** Regression Results for Experiment 2.

Model Term	Step 1	Step 2	Step 3
Charity framing	26.23	23.75	21.88
Social political ideology	3.39	2.85	−5.65
Charity framing*Social political ideology	−23.77**	−24.47*	−8.86
Perceived politics of the charity	–	9.04*	0.30
Perceived politics of the charity*Charity framing	–	–	6.25
Perceived politics of the charity*Social political ideology	–	–	8.11***
Age	2.07	1.29	1.32
Education	9.75	12.51	14.20
Age*Charity Framing	−0.32	0.19	0.13
Education*Charity Framing	13.18	−4.46	−5.64

*Note.* The 3-way interaction between charity framing, social political ideology, and perceived politics of the charity was tested initially but was not significant and was subsequently dropped from the model. Social political ideology and perceived politics were measured on a scale of −3 (*strongly conservative*) to 3 (*strongly liberal*) and charity framing was dummy coded as community (*0*) or religious (*1*). All statistics reported are unstandardized regression coefficients and should be interpreted in the context of these scales. Significance tests for *t* statistics associated with unstandardized regression coefficients are reported as *p* values:

* <.01,

** <.001,

*** <.0001.

**Figure 2:**
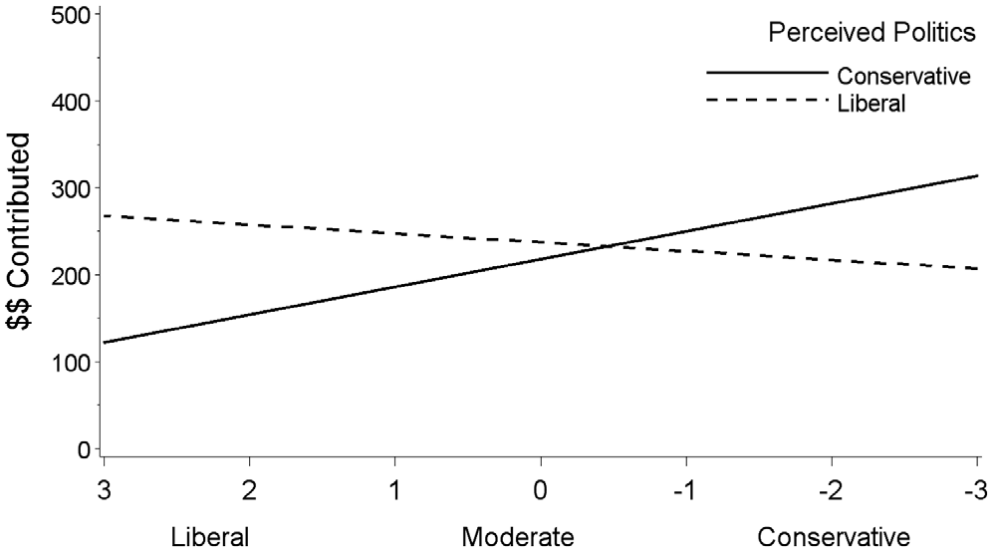
Perceived political belief similarity predicts favoritism for Christians. Regression analysis predicting the number of dollars contributed to the Community Service Center Charity from social political ideology and perceived political position of the charity.

To investigate the interaction between social political ideology and perceived politics of the charities, we coded the perceived politics of the charities as either conservative (collapsing *slightly*, *moderately*, and *strongly conservative*) or liberal (collapsing *slightly*, *moderately*, and *strongly liberal*) and examined correlations between participants’ social political ideology and contribution when the perceived politics of the key charity was either conservative or liberal. As seen in Figure 2, when the key charity was perceived as liberal, liberals gave more than conservatives, *b*(311) = 10.11, *p* = .051, *r* = .11, and when the key charity was perceived as conservative, conservatives gave more than liberals, *b*(383) = −31.91, *p*<.0001, *r* = −.36. Therefore, liberal Christians appear to have resisted favoring the religious charities because they perceived them to be relatively conservative, while conservatives favored them for the same reason. This provides evidence for the claim that in the context of religious identities, belief similarity cues favoritism.

#### Participants did not report awareness of belief similarity influencing favoritism

Participants reported that they were not thinking about the political position of the charities when they made their contributions (*M* = 1.50, *SD* = 0.90; scale range was 1 = *Not at all* to 5 = A *great deal*), that it did not influence their contributions in general (*M* = 1.61, *SD* = 0.98), or to the key charity in particular (*M* = 1.67, *SD* = 1.07). In fact, 66% of participants reported that their perceived politics of the key charity (across secular and religious framings) had no impact at all on their charitable giving. To test for the accuracy of these reports, we tested a model with main effects of perceived politics of the charity, social political ideology, charity framing, and their 2-way interactions predicting favoritism in this subsample of participants who reported no influence of perceived politics on their contributions (*n = *535). The interaction between perceived politics and social political ideology predicted contribution, *b* = 6.24, *CI* = 1.58, 10.90, *t*(525) = 2.63, *p* = .009, even in this restricted sample who reported that perceived politics was *not at all* influential. This interaction was also significant in the subsample of participants who reported that they were influenced by their perceived politics of the charities (*n* = 281), *b* = 10.37, *CI* = 5.11, 15.62, *t*(271) = 3.89, *p* = .0001. Some people accurately reported that they were influenced by the perceived political ideology of the charities, but the majority of them were not aware – or were not willing to report – this influence.

## General Discussion

In two studies, political ideology moderated ingroup favoritism for religious groups in a charitable giving task. Conservative Christians favored religious ingroups more than liberal Christians did. In fact, by giving more to a secular framed charity than religious or Christian-framed charities, liberal Christians’ pattern of favoritism resembled liberal agnostics and atheists more than conservative Christians. Further, Experiment 2 provided evidence that conservative Christians favored religious charities, and this was mediated by their perception of religious charities as conservative. Liberal Christians also perceived religious charities to be conservative, and this predicted their lack of religious group favoritism. Moreover, most participants reported no awareness of this influence.

These findings add to the literature demonstrating that conservatives show more ingroup favoritism than liberals do [7]–[9], [11]. However, the finding that liberal Christians resist favoring their religious ingroup is unique. Further, this reduced religious group favoritism among liberal Christians could not be accounted for by the strength of religious identity (Experiment 1) or the salience of their religious identity (Experiment 2), two factors that are known to influence group favoritism according to social identity and self-categorization theories [2]–[3]. We suggest that perceived belief similarity is an additional factor contributing to the presence or absence of ingroup favoritism, at least in the context of religious identities.

### Belief Similarity Influences Group Favoritism

Religious belief systems are associated with conservatism [16], [18], so liberal Christians find themselves in a strange position – their personal beliefs are liberal, but their religious ingroup beliefs are relatively more conservative. In the current studies, this conflict between personal and ingroup beliefs predicts decreased group favoritism. This supports previous research that finds that similarity of beliefs increases ingroup favoritism and attraction to ingroup members [28]–[29]. These findings are also consistent with Sani and Todman’s [30] schism model suggesting that group members who feel that their group has adopted a position that is misaligned with their values may withdraw and join a subgroup. However, in our case, the differences between liberal and conservative Christians hold even when accounting for the strength of identification with the religious group. Together, these findings suggest that belief similarity contributes to ingroup favoritism.

If liberals perceive their religious ingroups to be conservative, why identify at all with religious groups? Disidentification with religion is a possible reaction to belief dissimilarity. In support of this idea, Hout and Fisher [15] argued that because religion is associated with conservatism, liberals and moderates have been leaving the church, but retain their internal religiosity. Of course, not all liberals have abandoned religion. Instead of abandoning their religious identities entirely, some religious liberals might retain their religious identities and reconcile the conflict between their personal liberal beliefs and the conservative beliefs of their religious ingroup by resisting favoritism for religious ingroups. Perhaps liberal Christians’ decreased favoritism for religious groups observed in the current studies suggests that they will eventually disidentify entirely with their religious groups.

Belief similarity may play out in interpersonal contexts similar to how it functions in intergroup contexts. If belief dissimilarity occurs with family members or coworkers, it may not be desirable or even possible to end the relationship just the same as it may not be desirable or possible to leave a religious group, but it may be possible to avoid allocating resources or lending favors to the person. Future research can investigate the impact of belief similarity in interpersonal contexts. Future research can also investigate additional processes that result from belief (dis)similarity. For example, perceived dissimilarity between self and ingroup may cue trustworthiness of the group or other members of the group. This may impact group cohesion and eventually lead to the dissolution of the group or parts of it.

### Limitations and Alternative Explanations

The absence of an outgroup comparison in this study introduces the possibility that liberal Christians viewed a secular charity named the “Community Service Center” as more of an ingroup than the charity when it was framed as religious (e.g., “Christian Community Service Center”). This would require some additional nuance in interpreting our findings. If liberals have a more global social identity and prefer to think of all of humanity as their ingroup [47], they might favor groups that appear more inclusive, like “the community,” compared to exclusive, like religious groups. Conservatives’ moral concerns about ingroup loyalty serve to bind groups together [10] suggesting that conservative ideology contains a propensity toward “groupishness.” If conservatives are more “groupish” than liberals, they might prefer more exclusive groups to inclusive groups. To parse between these accounts, future research could again manipulate the ingroup status (whether the ingroup is described as religious or not) and also the inclusivity (whether the group is described as helping all people or only a specific group of people). If liberal Christians prefer inclusive groups, they should favor the group that helps all people, regardless of whether it reflects their religious ingroup or not. However, if liberals resist favoring religion exclusively because of belief dissimilarity, then they should disfavor religious groups, regardless of their inclusivity. It is conceivable that both mechanisms are operating to influence favoritism in the current studies.

Another possible alternative explanation to our interpretation is that instead of cuing belief similarity between self and religious (or community) group, belief similarity cued political group membership. If this account were true, then the evidence demonstrates a conflict between multiple identities (religious and political ingroups) rather than between personal beliefs and group membership. In this account, the findings observed here would reflect Christian liberals’ favoritism for the liberal political ingroup rather than a lack of favoritism for their religious ingroup. We believe that this explanation is less likely than our account because religious group membership was made explicitly salient in Experiment 2 – we manipulated the salience of religious identity prior to completing the charity donation task. Still, perceived belief similarity, not religious ingroup membership, influenced favoritism.

In our studies, the key charity was the only one that was manipulated to denote ingroup membership. This introduces the possibility that the interaction between the charity or its particular mission may have impacted the results. Also, given that only very short descriptions were given about each charity, familiarity with any of the charities – including religious charities – may have impacted contributions. However, the filler charities were real and the target charity was fictional across all experimental conditions, so it is unlikely that familiarity could account for the experimental effects. Nonetheless, future research extending the dependent variable to other formulations would strengthen the present interpretation.

Finally, our focus on Christianity as the religious ingroup may have influenced liberals’ lack of religious ingroup favoritism, and may not extend to other religious traditions. After all, conservatism is more strongly related to orthodox religious denominations than progressive ones [16], [48]. Future research can extend these findings to other religious groups that are more progressive on average than Christianity, such as Judaism [48]. Also, more orthodox denominations *within* Christianity (e.g., Mormonism) or *within* Judaism (e.g., orthodox Judaism) should be perceived as more conservative, and therefore elicit even less favoritism from (relatively more) liberal members than in the current context.

### Conclusion

People tend to perceive similarity between themselves and their ingroups [25]–[26], [28]. Such processes facilitate self-concept clarity and connection with ingroups. But, people are not completely ignorant to differences, especially when those differences are vivid – such as the belief systems of one’s religion compared to one’s own beliefs. Our findings suggest that the degree of perceived belief similarity influences ingroup favoritism in addition to other factors such as strength of identification with the ingroup and the salience of the ingroup. Perceived belief similarity may be a particularly important factor in group memberships like religion, where the belief system is a central component of the group composition and meaning.
